# Observations on Functional Disorders of the Spinal Cord, and Their Connexion with Hysterical, Nervous, and Other Diseases; Illustrated by Cases, Selected Chiefly from the Reports of the Pallas-Kenry and Currah Dispensaries

**Published:** 1830-03

**Authors:** Wm. Griffin, D. Griffin

**Affiliations:** Limerick.; Limerick.


### FUNCTIONAL DISORDERS OF THE SPINAL CORD.

*Observations on Functional Disorders of the Spinal Cord, and their Connexion with Hysterical, Nervous, and other Diseases; illustrated by Cases, selected chiefly from the Reports of the Pallas-Kenry and Currah Dispensaries.* By WM. GRIFFIN, M.D. and D. GRIFFIN, M.R.C.S. Limerick.

(Continued from page 110.)

THE effects of mental emotion on the heart and arteries, the irregular, or violent, or preternaturally weak, or suspended action which they induce, through the medium of the spinal cord, might have led to the knowledge of a new train of symptoms dependent on irritation. As these are affections of the involuntary muscles, the power of which is supposed to depend on the ganglionic nerves, we were, in our earlier attention to the subject, much perplexed what to attribute to diseased states of the ganglia, and what to the cord. Assuming, from Dr. Philip's experiments, "that the heart is not only as easily stimulated through the brain and spinal marrow as the muscles of voluntary motion are, but that it may be stimulated through them in the newly-dead animal for a considerable time after these muscles can no longer be influenced in this way; proving that the ganglions oppose no obstacle to the influence of the brain and spinal marrow being extended to the muscles of involuntary motion," we saw no difficulty in concluding that disturbed states of the spinal marrow may produce such affections. We felt, however, that it was highly probable they might equally result from diseased ganglia, and even be accompanied by the tenderness of the corresponding portion of the medulla. However, as our knowledge of the functions of the sympathetic and its ganglions is as yet too obscure to permit any certain inferences, it eventually seemed most prudent to assume as affections of the cord all such as were attended by spinal tenderness, and to give to the ganglia those which evinced no such symptoms. The danger of erroneous conclusions was thus at least limited. There could be no doubt of the influence of the cord on the involuntary muscles, nor that this influence might be excited by irritation of certain portions of it; while it seemed altogether a gratuitous supposition that spinal tenderness is ever induced by diseases of the ganglia. It may be quite true that it is so, but we do not seem to have any proof of it.

We are the more anxious to insist on this point, because it is a matter of no small importance to have any mode of distinguishing between affections of these nervous masses and those of the cord; and in a treatise on this subject which has just come into our hands, written by Mr. Teale, of Leeds,* it is freely assumed that affections of all parts, whose functions are chiefly dependent on the sympathetic, are the result of disease in the ganglia of that nerve. It seems needless to repeat what Dr. Wilson Philip has so fully explained, that although the stomach, intestines, &c. are wholly dependent upon the ganglionic system for the exercise of their functions, they are strictly subject to the influence of the brain and spinal marrow; and we are hence at a loss to conceive why the spinal tenderness should be mentioned by Mr. Teale as an argument for ganglionic disease, when the most obvious conclusion would be that it indicated an affection of the cord. It seems difficult to explain, too, why, in some cases exhibiting intense symptoms of ganglionic disease, this tenderness should be wholly absent. It may, indeed, be said, with great truth, that many instances of spinal disease occur in which the tenderness is very limited, and nowise proportioned to the symp-

* Our remote situation from the metropolis precluding the possibility of an early sight of new publications, we have only just received Mr. T. P. Teale's excellent little work on Neuralgic Disease. Although a little surprised to find ourselves so largely anticipated, even to very arrangement, it was no slight gratification to find our views fully confirmed by this gentleman's observations. We have in some instances hesitated in going so far as Mr. Teale did, while in others our investigations have tempted us beyond him.

toms. Of such it is only fair to admit a strong probability that the ganglia are the true seat of the disease.

It appeared from Dr. Philip's experiments, that to produce any powerful effect on the heart and blood-vessels, it was necessary to stimulate a large portion of the brain and spinal marrow. The same fact is observable in the diseases of irritation, as most severe cases of palpitation, syncope, &c. not occasioned by organic disease, are marked by universal tenderness of the spine. We have nevertheless often met with these symptoms connected with tenderness of minute points, especially of the cervical portion; and whether this be owing to a peculiar sensation occasioned at the tender part, and acting on the whole cord, or, in the latter case, to an affection of the pneumo-gastric nerves, which have so considerable a share in forming the cardiac, or finally, to an affection of the cervical ganglia, it is difficult to determine. For illustrations on this subject, instead of extracts from our note-book, we might perhaps refer with more advantage to an essay by Dr. Darwall, of Birmingham, of which we have only been fortunate enough to see an interesting notice. Nothing can be more accurate than his delineation of these affections. We shall only venture to offer two or three cases, commencing with one resulting from the irritation produced in the cervical cord by an injury of the external ear.

XXII. James Casey, a smith, had part of his ear bitten off in a quarrel. The inflammation and soreness was so great that he could not sit up in bed, and, though a strong man, generally fainted when the sore was dressing. This did not excite much surprise at first, as it was attributed to the tenderness of the wound in a peculiarly sensitive habit; but when it began to heal, and all extraordinary soreness had worn away, it seemed very remarkable that he should still continue subject to sudden sinkings or lownesses, closely approaching to syncope. There appeared an extravagant disproportion between the apparent debility or nervous depression, and the trifling nature of the wound. When the lowness came on, he was always terrified by the apprehension of dying, and was obliged instantly to have recourse to wine and stimulants for relief, which, as he had no thirst nor heat of skin, were not forbidden. From the resemblance which these fits bore to the sinkings which are sometimes observed, in hysterical habits, in females after delivery, irritation of the cervical portion of the spinal marrow was suspected. On examination, there was found very great tenderness at the third and fourth cervical vertebræ, particularly acute at the right side. As the wound was now healed; and the disposition to fainting was much less frequent, it was thought unnecessary to institute any local treatment: attention to the bowels, and the volatile tincture of valerian, with camphor mixture, completed the cure.

XXIII. Mrs. ——, a lady aged forty-eight, was awoke in the night by a sensation of weight and constriction across the chest; pain at the ensiform cartilage and violent palpitation, followed by fits of sinking or fainting, with apprehension of dying. The palpitation was always brought on to a distressing degree when she chanced to turn on her left side. These symptoms continued to recur for some days, but were very much relieved by mild purgatives and antispasmodics: she was, however, now seized with acute pains in the neck, arms, chest, and sides; and, on examination, there was found tenderness of the first and second cervical vertebræ, and of the seventh or eighth dorsal. All these symptoms readily disappeared on the application of a blister to the latter place; and her usual health was restored by a continuation of the antispasmodics, with tonics. The attack seemed to have originated in fright and mental anxiety, and was readily reinduced, though in a slighter degree, by any new distress, for several months afterwards.

Although we suppose, in these cases, that the primary disease exists in the cord, the ganglia are necessarily implicated. It is on them, and through them, the spinal irritation exerts its influence; and we may have the upper or lower ganglia affected, according as the irritation shifts its place in the spine. As an instance of this, we have been, within these few days, sent for by the lady whose case is just detailed, and found her labouring under a new train of symptoms: she had been seized, two or three times during the last week, with a sudden rush of blood to the head, which seemed to commence at the clavicles, and pass up in the course of the carotids. There was a momentary faintness, or tendency to insensibility, with loss of power of the arms; and she complained of occasional pain at the crown of the head and brow, sometimes occurring suddenly, and attended by stiffness and tenderness of the muscles at the back of the neck, especially at the right side; she had also slight cough, and an internal soreness of chest, which she compared to the sensation experienced when a blister is taken off. She has had also a return of the palpitations at night. There can be no doubt of these symptoms yielding to the usual treatment.

XXIV. James Hanly, aged twenty-eight years, a labourer, about six months since, after severe labour in drawing loads of manure on his back, felt pain between the shoulders and in the back of the head, with continual drowsiness; could not keep himself awake, and sometimes fell into a state of insensibility, in which he usually lay for some minutes. This was especially apt to occur to him in bed, coming over him, as he expressed it, "like a dream." He had a continual disposition to yawn and stretch himself, which sometimes induced him to hang by the arms from the branch of a tree; but this always brought on the pain between the shoulders and at the back of the head. He lost all appetite, and was frequently attacked with racking pain or cramp in the stomach and sides. He gave up eating in the evening, as he invariably found that it brought on the pain. After some weeks, the headach became more distressing, and he began to be attacked by palpitations, which were soon constant and very troublesome, accompanied by tremblings or shiverings, and by universal throbbing of the arteries. He felt pulses, he said, in every part of his body. The palpitation often occurred in sudden paroxysms; the heart beating as if it would burst the walls of the chest. They frequently extended to the descending aorta, and were felt most distressing at the back. When attacked in this way, he was always obliged to spring from the bed, and walk about the room, until the throbbing abated. In its greatest violence, it was sometimes accompanied by pain or uneasiness in the cardiac region, which passed suddenly to the throat and head, and left him in a state of insensibility. After some months had elapsed, the headach became so distressing, and the pain of the stomach so severe, that it was found necessary to take blood largely twice from the temporal artery within a short period. From this considerable benefit was derived; but the palpitations still continued, often waking him up at night, and terminating in a protracted fit of insensibility, somewhat like syncope, except in the flushing and heat of the face and brow. These usually commenced with general shivering, followed by pain between the shoulders, profuse perspiration, palpitation, and insensibility. He attributed his illness to lifting heavy loads, but thought it might possibly have some connexion with a fracture of the skull which he had received four years since. He was, however, in good health from the time he received the injury until within the last half-year, when his present illness commenced.

The treatment consisted of the bleedings already mentioned; the constant use of the tartarized antimonial ointment to the spine; the Pil. Aloet. cum Assafœtid., with volatile alkali in camphor mixture; and one or two blisters to the back of the neck. There was a perfect recovery in three or four weeks. The spinal tenderness, which was extremely acute in all the cervical and in the seventh and eighth dorsal vertebræ, gradually declining in proportion to the amendment in the more distressing symptoms of the complaint.

In the cases already detailed, especially in our first, it will be recollected that many or all of these symptoms were present, and we shall find them forming a link in the chain of morbid phenomena in almost every severe one which we may have to offer as we proceed. It is necessary again to call the reader's attention to the spinal tenderness which, from its almost invariable presence in such cases as occur to us, we believe may be found in the very earliest stages of all those dreadful fits of palpitation, angina, syncope, &c. dependent on dyspepsia and hysteria. It can matter little whether it be characteristic of a primary affection, created we know not how, or of one originating in the mental, the digestive, or generative organs, if it is capable of becoming independent, and reacting on the system. The only question of importance appears to be, is it casually or ever induced by organic disease of the heart or large vessels? or is it not rather the precursor of these?

The former point we have had but few opportunities of ascertaining; but Mr. Teale states that he has met with some instances of it. We have nevertheless strong grounds for believing that, in the majority of cases, functional disorder of the circulation, and its attendant spinal tenderness, precedes change of structure. The well-known observation of Corvisart, that palpitations and structural affections of the heart increased much during the dreadful period of the French revolution, may be mentioned in illustration of this; as we must suppose the passions of the mind can only act in the first instance on the functions of parts. We have already observed that spinal tenderness does not accompany inflammatory, and we believe it may be said organic, disease in general; and if we could offer it as a certain diagnosis in these as in other affections, it would be very satisfactory; but this would be deducing more from it than, in such obscure complaints, any one character can ever possibly give, and, at all events, must be left to the determination of more extensive observation.

It seems much if its presence materially assists in the diagnosis, and we can conceive cases in which it may decidedly pronounce it, even where the stethoscope, with the most practised ear, has failed.

It may seem, perhaps, extravagant or paradoxical to suppose that functional disorder could imitate the physical signs of organic change; and yet this we are far from thinking impossible.* Cases have occurred to us in which there appeared to be the utmost moral certainty of organic disease, and which, notwithstanding, got completely well in the course of some months or years, without having been materially benefited by any medical treatment. Dr. Abercrombie relates some such; two of which, if we may reason from the treatment and result, originated in a gouty or rheumatic diathesis. It is not improbable that spinal tenderness would have been found in all of them. Similar ones are related by Cullen and Mason Good; and there can be little doubt but the singular affection of which the late Dr. Bateman eventually died was of this description.

With cases of violent, or irregular, or depressed action of the heart, may be considered similar affections of the arterial system, which will very generally be found to depend on that morbid condition, of a greater or less extent, of the cord, called irritation. Preternatural throbbing of the temporal and carotid arteries will usually be found connected with cervical tenderness, while the pulsations of the aorta in the epigastric region are more probably referable to irritation of the upper dorsal. We speak in this qualified way, because, in all the cases we have met with, there existed tenderness of the whole or the greater part of the spine. Before functional disorders of the cord became a subject of investigation with us, we were often much alarmed and perplexed by these epigastric pulsations, and, in cases presenting many marks of an exquisitely nervous diathesis, were imperfectly contented to assume, with Dr. Baillie, Hunter, and others, that they were not dependent on organic disease; but we now have, it is hoped, in the spinal tenderness, a certain diagnostic, by which apprehension may be at once allayed. Even if it be true that it sometimes is to be met with in aneurism of the aorta, we suspect it would not, for a length of time, give rise to those peculiar nervous symptoms indicative of spinal irritation,

* Laennec, who ought to be excellent authority on this subject, asserts that, in functional disturbance of the heart, the stethoscopic signs attendant on organic affections may sometimes be detected, and instances especially the *bruit de soufflet*.

which may always be detected at the very earliest period of that affection. A more perplexing case may be imagined in pure disorder of the ganglionic system, on the supposition that it does not occasion spinal tenderness; as here we should have the violent palpitation, as in aneurism, without any mark of affection of the cord; but it is to be recollected that the great sympathetic connects and combines so many parts, by the intimate interweaving of its numerous threads, that no one portion of it could be seriously affected without inducing general disturbance of the abdominal or thoracic viscera. In any symptoms of an hysterical or nervous character, palpitation from ganglionic irritation could not exist with symptoms so few and uncomplicated, and so analogous to what we might associate with physical effects, as those arising from aneurism.

Instances of these affections of the arteries may be found as occasional symptoms, in almost every severe case of spinal irritation which we shall have occasion to quote.

Perhaps no fitter opportunity may occur to offer a few words relative to the disease called angina pectoris, which we have had every reason, with M. Laennec, to regard as a neuralgic affection, and, as far as our experience has gone, connected with cervical tenderness. We have not had the good fortune lately to meet with a pure or intense case; but in its less aggravated form, and often complicated with other symptoms, it has frequently fallen under our notice. It was long a distressing affection in the first case detailed in these papers. Mr. Teale has given some very interesting ones, which we are glad to find fully confirmatory of these views.

In concluding this short notice of affections of the circulating system, we would offer one observation. Previous to the time of Corvisart, those resulting from its organic change were little understood, and believed to be very uncommon. The most obvious cases were treated as nervous disorders. The study of morbid anatomy at length produced a total change in medical opinion, and it was soon believed that most or all of them depended on absolute physical changes. As a consequence of this, innumerable patients, who were suffering from dyspepsia, gout, or rheumatism, were put under the severest discipline, and thrown into a state of miserable dejection, by pronouncing their cases organic. Frequent error has now led us to tread back our steps, and we would only beg to remind the reader that, the stronger our reasons may be for doing so, the greater the risk of our again, like the older pathologists, overlooking organic cases. We know that those called nervous or symptomatic are infinitely the most numerous, but we are also fully impressed with the conviction that instances of altered structure are of daily occurrence.

[To be continued.]

---

### PERFORATION OF THE STOMACH.

